# Estimation of Accuracy of B-Mode Sonography and Elastography in Differentiation of Benign and Malignant Lymph Nodes With Cytology as Reference Standard: A Prospective Study

**DOI:** 10.7759/cureus.14147

**Published:** 2021-03-27

**Authors:** Rahul Arkar Rajendra, Rajesh Kumar Varatharajaperumal, Rupa Renganathan, Venkatesh Kasi Arunachalam, Pankaj Mehta, Mathew Cherian

**Affiliations:** 1 Radiology, Dr. D.Y. Patil Medical College, Hospital and Research Center, Pune, IND; 2 Radiology, Kovai Medical Center and Hospital, Coimbatore, IND

**Keywords:** ultrasound, sonoelastography, cervical lymph nodes, lymphadenopathy, fine needle aspiration cytology

## Abstract

Purpose: To prospectively estimate the reliability of B-mode ultrasonography and sonoelastography in differentiating benign and malignant cervical lymph nodes with cytological findings as to the reference standard.

Materials and Methods: A total of 50 patients referred for sonography for enlarged cervical lymph nodes were included in the study. They were subjected initially to B-mode ultrasonography and sonoelastography and later underwent fine-needle aspiration cytology (FNAC) in the same sitting. Sensitivity, specificity, and accuracy were compared.

Results: Out of 50 cases, 33 were males, and 17 were females. On B-mode ultrasonography, 15 enlarged cervical lymph nodes were benign-looking and 35 were malignant-looking. When studied on elastography, 12 were benign-looking and 38 showed features of malignancy. However, when studied histopathologically, 18 were benign and 32 were malignant. The sensitivity, specificity, and diagnostic accuracy were compared, and the results were better in sonoelastography than B-mode ultrasonography. When both B-mode and sonoelastography were combined, an increase in the sensitivity for differentiation was achieved. However, a decrease in specificity was noted when both modalities were combined in our study, probably due to a significant number of patients with tuberculous cervical lymphadenopathy.

Conclusion: In countries like India, where granulomatous infection like tuberculosis is prevalent, the combination of sonoelastography with B-mode ultrasonography has decreased specificity in the differentiation of benign and malignant cervical lymph nodes, and histopathology is always needed for the final confirmation of diagnosis. The decreased specificity on elastography is attributed to simultaneous coexisting inflammation and fibrosis in chronic granulomatous lymphadenopathy.

## Introduction

Metastatic cervical lymph nodes are common in patients with head and neck, as well as non-head and neck cancers [1-3]. Cervical lymph nodes are common sites of lymphoma involvement, tuberculous lymphadenitis, and other benign lymphadenitis such as Kikuchi disease, Kimura disease, and Rosai-Dorfman disease [4,5]. Appraisal of the nodal condition is requisite in patients with head and neck carcinomas as it forecasts the prognosis and helps in selecting treatment alternatives [6-8].

Both medical and dental professionals worldwide face problems in the early perception and diagnosis of such cancers [9]. Fortunately, there has been a substantial increase in the development of probable cancer screening and case finding tools [10]. At times metastatic cervical lymphadenopathy is the first symptom in patients with malignancies of the head and neck, lung, breast, and other sites. Hence, differentiation between benign and malignant lymphadenopathy is essential, and one of the differentiating indicators is lymph node hardness or elasticity [11].

Dr. George Ludwig in the United States of America first used ultrasound in medical applications more than 50 years ago. Since that time, ultrasonography devices, and methodologies have continuously progressed and become more important as tools in diagnostic medicine [12-14]. In the last few years, a significant breakthrough was brought by the inception of sonoelastography used to accurately identify the character of pathoanatomical changes and their stages [15,16]. The three main types of sonoelastography are strain/compression elastography, shear wave elastography, and transient elastography.

Sonographic elastography works in the following steps: first, elastography receives digitized radiofrequency echo lines from the tissue; second, it gives slight compression to the tissue by the transducer along the radiation axis to make some displacement; and third, it receives a second post-compression digitized radiofrequency echo line from the same tissue. Then, the data undergo processing, and ultimately, an elastographic image appears on the monitor [17].

Sonoelastography is used to differentiate hard and soft tissues. It measures tissue reaction to stress, calculates strain values, and typically displays this information as a color overlay on a conventional B-mode ultrasound image. The stiff structures are displayed as blue while the more easily deformed tissue are displayed as red, green indicating an average value of strain [18].

The objective of this study was to estimate the accuracy of strain elastography in differentiating benign and malignant etiology in patients with enlarged cervical lymph nodes along with histopathologic nodal findings.

## Materials and methods

In this study, 50 consecutive patients from the age group 9 years to 85 years referred for sonography for enlarged cervical lymph nodes were enrolled. The study included 33 males (66%) and 17 females (34%).

The institutional ethical committee approved this study, and written informed consent from each patient was received. All the patients were examined with Voluson-E8, ultrasound equipment. Then, the enlarged lymph nodes were evaluated with fine-needle aspiration cytology (FNAC).

The patients with tender lymph nodes, ulcerated lesions, not willing to consent, lost to follow-up, and those whose histopathology reports were not available/negative cytology were excluded from the study.

Procedure

B-mode ultrasonography and sonoelasotography scans were performed transcutaneously on all patients. Patients underwent B-mode Ultrasonography in the supine position with the neck in the extended position. Enlarged lymph nodes were assessed to record short-axis diameter, short axis to long axis diameter ratio, hilum, echogenicity, calcifications, and vascularity. When more than three parameters were positive, the node was classified as malignant.

**Figure 1:**
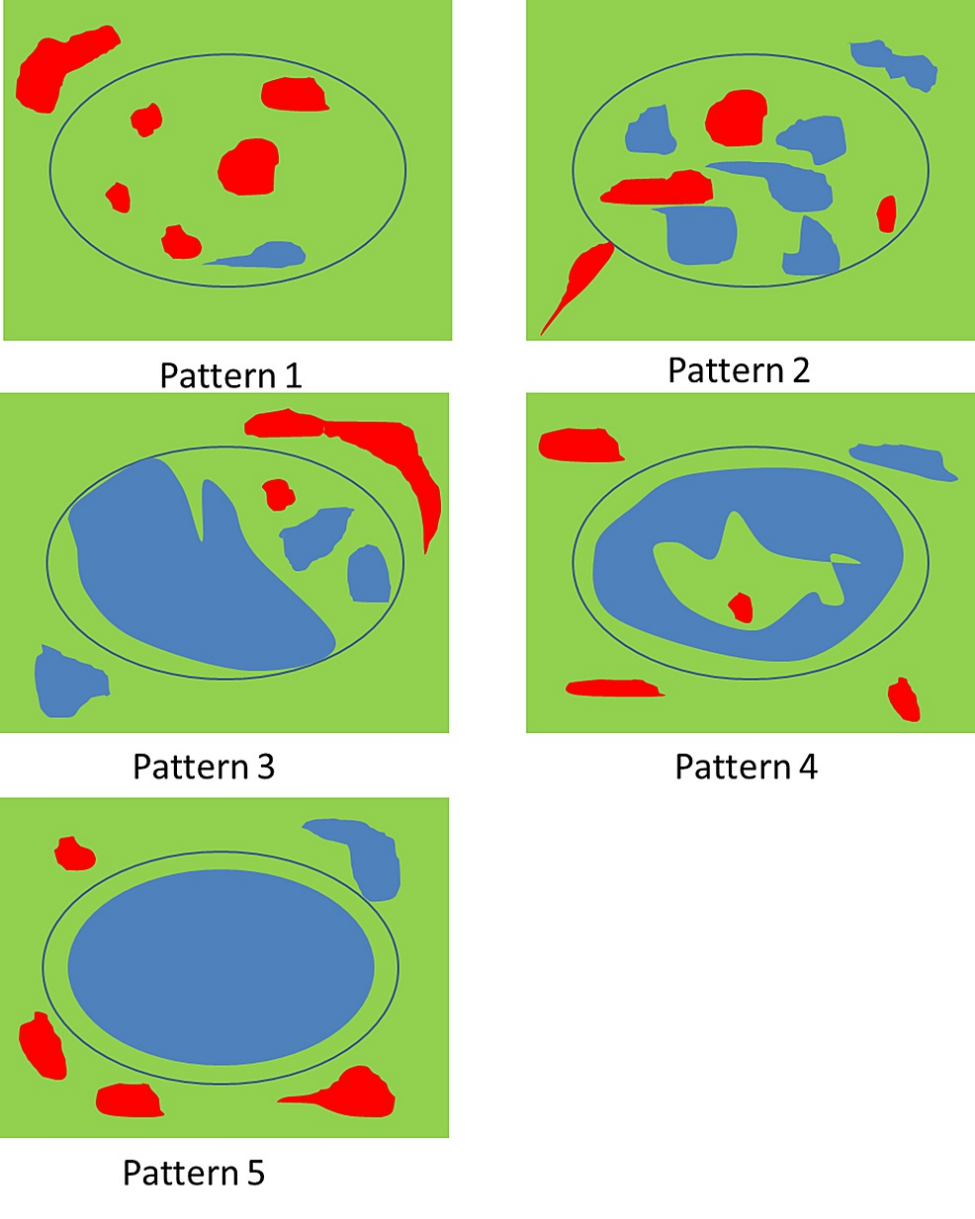
Elastographic patterns.

After the B-mode ultrasonography, the nodes were evaluated for stiffness using strain type of sonoelastography. The nodes were assessed with a linear probe. The lymph node was compressed and slowly released; the green color on the sidebar indicated adequate compression. Inadequately compressed images that showed red or yellow color were discarded. The final sonoelastographic pattern of lymph nodes was evaluated and categorized into five groups (Table 1) [19], and images were recorded (Figure 1). Then, the patients were followed up by histopathology.

**Table 1:** Patterns and scoring system on elastographic findings.

Pattern	Score	Description	Elastographic diagnosis
1	2	Absent or very small blue area(s)	Reactive
2	4	Small scattered blue areas, total blue area <45%	Reactive
3	6	Large blue area(s), total blue area ≥45%	Malignant
4	8	Peripheral blue area and central green area, suggesting central necrosis	Malignant
5	10	Blue area with or without a green rim	Malignant

## Results

**Figure 2:**
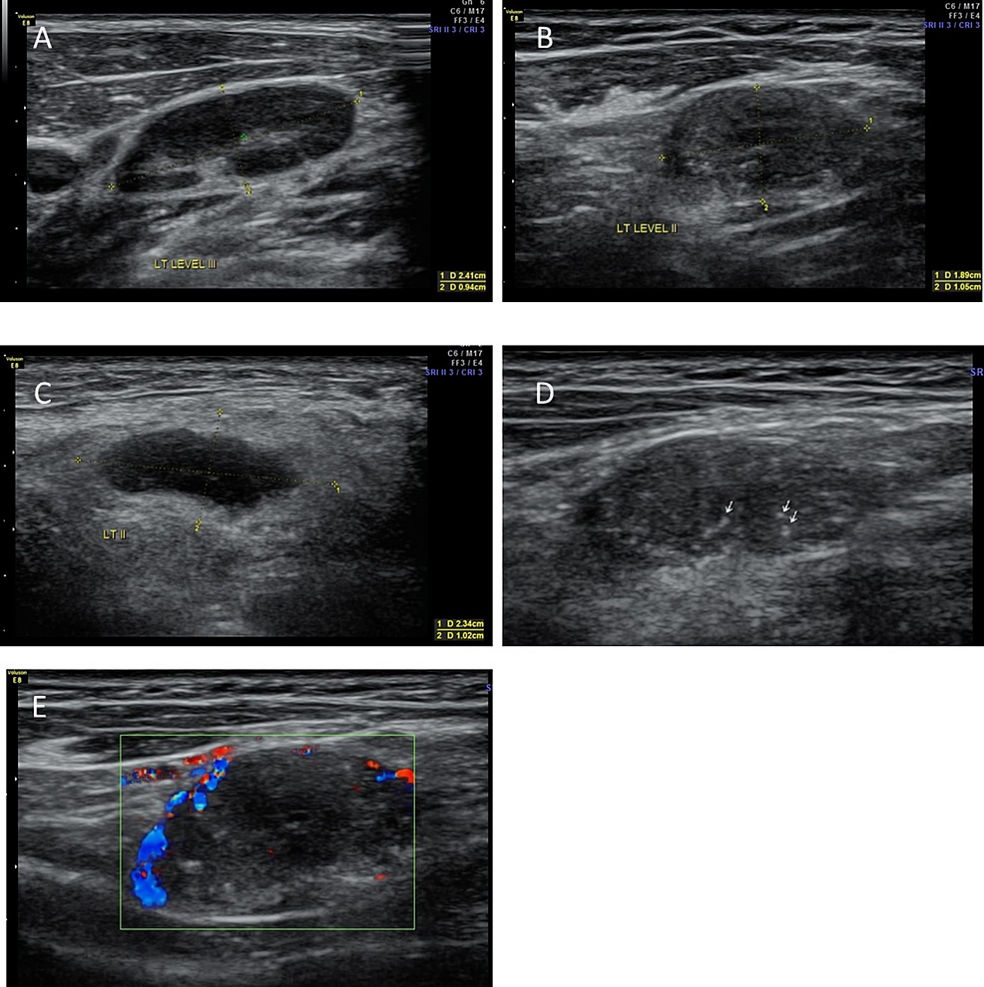
B-mode (A-D) and Doppler (E) ultrasonography images of cervical lymph nodes. (A) B-mode ultrasonography showing short-axis dimension more than 0.8 cm and normal hilum; (B) B-mode ultrasonography lymph node showing short-axis dimension more than 0.8 cm and short-axis to long-axis diameter ratio of more than 0.5; (C) B-mode ultrasonography showing enlarged hypoechoic lymph node with absent hilum; (D) B-mode ultrasonography showing calcifications (white arrows) in lymph node; (E) ultrasound doppler study showing predominantly peripheral vascularity.

**Figure 3:**
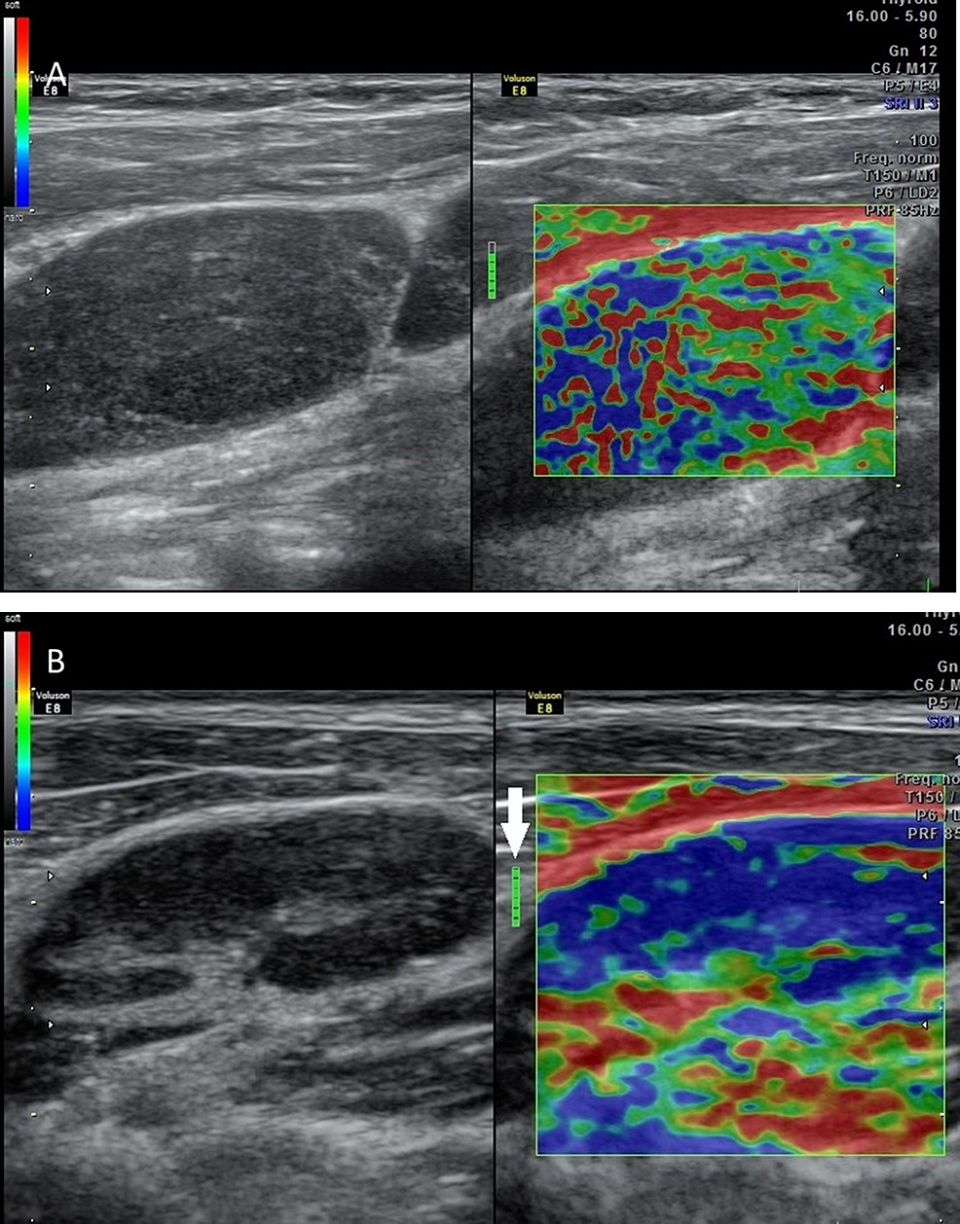
Sonoelastography images of cervical lymph nodes. (A) Ultrasound elastogram image showing pattern I suggestive of benign etiology; (B) ultrasound elastogram showing pattern IV suggestive of malignant etiology. The green colour in the feedback indicator (white arrow) indicates adequate compression.

The study comprised a sample size of 50 patients. B-mode ultrasound and sonoelastography findings (Figures 2 and 3) of enlarged cervical lymph nodes are shown in Tables 2 and 3, respectively.

**Table 2:** Characteristics of lymph nodes in B-mode ultrasound.

Imaging examination and characteristics	Number of patients	Percentage
B-mode ultrasonography		
Short-axis diameter		
≤8 mm	8	16%
≥8 mm	42	84%
Short- to long-axis diameter ratio		
≤0.5	10	20%
≥0.5	40	80%
Hyperechoic hilum		
Absent	34	68%
Present	16	32%
Echogenicity		
Abnormal	38	76%
Normal	12	24%
Micro-calcifications		
Absent	44	88%
Present	6	12%
Vascularity		
Hilar	17	34%
Peripheral/mixed	33	66%

**Table 3:** Characteristics of lymph nodes in sonoelastography.

Patterns	No of patients	Percentage
I	5	10%
II	7	14%
III	4	8%
IV	20	40%
V	14	28%
Total	50	100%

FNAC

The final diagnosis was confirmed with FNAC. The cytology turned out to be benign in 18 patients (36%). Among these, six patients (12%) had reactive hyperplasia, 6 (12%) had tuberculosis, and 3 (6%) had chronic non-specific lymphadenopathy. Acute suppurative inflammation was seen in three patients and necrotizing histiocytic lymphadenopathy (Kikuchi lymphadenopathy) in one patient.

The prevalence of malignant lymph nodes was 32/50 (64%). Among the malignant lymph nodes, 15 (30%) had primary squamous cell carcinoma, 9 (18%) had papillary carcinoma of the thyroid, 3 (6%) had lymphoma, 3 (6%) had adenocarcinoma, 1 (2%) with low-grade adenoid cystic carcinoma, and 1 (2%) was unknown.

B-mode sonography

Out of 50 cases, 15 (30%) were benign-looking, and 35 (70%) were malignant-looking on B mode ultrasonography. Among 18 (36%) cytologically proven benign lymph nodes, 10 (20%) were already reported as benign lymph nodes looking on B-mode ultrasonography, additional 5 (10%) were malignant. Among these, five false-positive benign cases on B-mode ultrasonography, one case of low-grade adenoid cystic carcinoma, squamous cell carcinoma, papillary carcinoma of the thyroid, and two cases of metastatic adenocarcinoma were included.

Among 32 (64%) cytologically proven malignant lymph nodes, 27 (54%) cases were already reported as malignant looking on B-mode ultrasonography. However, an additional 8 (16%) cases were false positive on B-mode ultrasonography, 2 (4%) had acute suppurative inflammation, 4 (8%) had tuberculosis, and 2 (4%) had reactive hyperplasia.

Sonoelastography

On evaluation, out of 50 cases, 12 showed features of benign etiology, and 38 depicted features suggestive of malignant etiology. Among 18 benign cytologically proven cases, 10 cases showed patterns I and II (reactive), while eight were diagnosed as malignant looking on sonoelastography. Among 32 cases of cytologically proven malignant cases, 30 cases were diagnosed as malignant looking on sonoelastography, 17 showed pattern IV, 11 showed pattern V, and 2 showed pattern III.

There were two false-negative cases, 1 had Classic Hodgkin's lymphoma, and 1 had low-grade adenoid cystic carcinoma. Additional eight cases were false positive on sonoelastography. These include five cases of tuberculosis (patterns III, IV, and V) and one case each of necrotizing histiocytic lymphadenopathy (Kikuchi lymphadenopathy), chronic non-specific lymphadenitis (pattern IV), and reactive hyperplasia (pattern V).

Diagnostic performance

The diagnostic performance of B-mode sonography and elastography was compared with FNAC. The sensitivity, specificity, positive predictive value, negative predictive value, and diagnostic accuracy were shown in Table 4.

**Table 4:** Comparison of diagnostic performance of B-mode ultrasonography, sonoelastography with histopathology.

S. No	Diagnostic performance	B-mode sonography	Sonoelastography	B-mode and sonoelastography
1	Sensitivity	84.4%	93.8%	96.9%
2	Specificity	55.6%	55.6%	33.3%
3	Positive predictive value	77.1%	78.9%	72.1%
4	Negative predictive value	66.7%	83.3%	85.7%
5	Diagnostic accuracy	74%	80%	74%

## Discussion

Cervical lymphadenopathy is a common day-to-day clinical finding. Cervical lymph nodes are one of the common sites for diseases like lymphoma, tuberculous lymphadenitis, and other benign lymphadenitis such as Kimura disease and Rosai Dorfman disease [1,4,5].

Ultrasound imaging is a dynamic, readily available, and patient-friendly technique instrumental in examining superficial structures [9]. Ultrasound imaging results in the rapid acquisition of images with minimal artifacts and can guide needle biopsies, making it a precise diagnostic tool [9,19]. Our study had conducted ultrasound imaging along with FNAC.

In 1991, Ophir et al. described a new ultrasound technique known as sonoelastography that computes tissue compliance characteristics [9,20]. It allows evaluation of elasticity distribution and shows differences in hardness between diseased tissue and normal tissue. This technique has promising results in the differential diagnosis and may validate as exceptionally useful in analyzing lymph nodes [9,21].

Our study also solicits elastography in examining cervical lymph nodes. Neck lymph nodes were easily approachable and efficiently compressed against the underlying anatomic structures. Elastography and relative tissue elasticity are color mapped into five groups. In this color map [22], blue indicates the hard area that indicates relative tissue hardness, which approves malignant involvement. Since long, various parameters were based on B-mode ultrasonography to differentiate benign from malignant lymphadenopathy are available in the literature [22-25]. Strain elastography is more operator-dependent than shear wave elastography [26].

The cut-off short-axis diameter used in our study was 8.0 mm, while the cut-off-axis diameter for level II lymph nodes was two times higher as for level III. This suggests that for evaluating cervical lymph nodes, a standard cut-off diameter should not be used at all levels. The study conducted by Alam et al. described the cut-off short-axis diameter of 8.0 mm [11].

Our study gave the best accuracy at the short-axis to long-axis diameter ratio of less than 0.5 for benign and more than 0.5 for malignant lymphadenopathy. These findings correlate with the round shape of malignant-looking lymph nodes in the study of Alam et al. [11].

The hyperechoic hilum of lymph nodes, whether present or absent, is considered an essential criterion for the differential diagnosis of cervical lymphadenopathy. In our study, this parameter showed the best accuracy for malignant-looking lymphadenopathy. However, benign-looking lymphadenopathy showed loss of normal hyperechoic hilum due to liquefaction associated with suppurative inflammation destroying the hilum. Similar findings were seen in the study conducted by Alam et al. [11]. Our study had assessed intranodal vascularity on Doppler ultrasound. It showed that benign lymph nodes had hilar vascularity while metastatic lymph nodes had peripheral or mixed vascularity. These findings correlate with the findings of Alam et al. [11].

The sensitivity, specificity, and diagnostic accuracy of B-mode ultrasonography in our study were 84.4%, 55.6%, and 74%, respectively. The results were similar to the study conducted by Alam et al. [11]. In their study, the sensitivity, specificity, and diagnostic accuracy were 98%, 59%, and 84%, respectively. The sensitivity and diagnostic accuracy in our study were lower than that in the study by Alam et al. [11]. This may be due to the fact that benign lymphadenopathy was categorized only on follow-up in their study. However, in our study, the final diagnosis was based on cytological findings. Our study had more tuberculosis, granulomatous inflammation, and lymphoma, whereas, the study conducted by Alam et al. did not encounter these cases.

Based on the patterns of elastograms in our study, sensitivity, specificity, and diagnostic accuracy were 93.8%, 55.6%, and 80%, respectively, compared with F Alam's study, 83% 100%, 89%, respectively. In a meta-analysis by Xie et al., the pooled sensitivity and specificity in differentiating benign from malignant lymph nodes were 85% and 86%, respectively [27]. Compared with Alam et al., our study showed higher sensitivity while lower specificity and diagnostic accuracy. This may be due to the study of benign lymph nodes studied by Alam et al., which was based on follow-up and not on histopathology, which might have given high specificity. When B-mode ultrasonography and elastography findings were combined to differentiate benign from malignant cervical lymphadenopathy, the sensitivity, specificity, and diagnostic accuracy were 96.9%, 33.3%, and 74%, respectively. However, it was 92%, 94%, and 93% in the study conducted by Alam et al. [11]. This shows that the differences may be due to the study of benign cases only on follow-up by Alam et al. [11]. The study by Alam et al. was similar to the study done by Zakaria et al. [28]. Our study had tuberculosis and chronic granulomatous inflammation cases, which had given false-positive results as metastasis, leading to lower specificity and diagnostic accuracy. Our results were similar to the study done by Teng et al. [29]. In their study, the sensitivity, specificity, and diagnostic accuracy of elastography was 88.40%, 35.10%, and 66.30%, respectively. In their study, 11 out of 16 tuberculous lymph nodes had given false-positive results with ultrasound elastography. The decreased specificity on elastography is attributed to simultaneous coexisting inflammation and fibrosis in chronic granulomatous lymphadenopathy [30]. The fibrosis component is responsible for a false-positive result on elastography.

## Conclusions

When combined with B-mode ultrasonography, elastography is a promising imaging technique and proves to be most reliable in the differentiation of benign from malignant cervical lymph nodes. Though elastography, when combined with B-mode ultrasound, increases the sensitivity and negative predictive value, a significant decrease in the specificity was noted in our study. This was most probably due to an increased number of granulomatous inflammation in our study. The decreased specificity on elastography was attributed to simultaneous coexisting inflammation and fibrosis in chronic granulomatous lymphadenopathy with fibrosis component responsible for a false-positive result. So in countries like India, where there is an increased prevalence of granulomatous diseases like tuberculosis, it is difficult to rely on ultrasound (B-mode and sonoelastography) alone. It is always best to use histopathology for the final diagnosis as this alters the treatment course.
